# MOFSocialNet: Exploiting Metal-Organic Framework Relationships via Social Network Analysis

**DOI:** 10.3390/nano12040704

**Published:** 2022-02-20

**Authors:** Mehrdad Jalali, Manuel Tsotsalas, Christof Wöll

**Affiliations:** Institute of Functional Interfaces (IFG), Karlsruhe Institute of Technology (KIT), Hermann-von Helmholtz-Platz 1, 76344 Eggenstein-Leopoldshafen, Germany; mehrdad.jalali@kit.edu

**Keywords:** metal organic framework, social network analysis, centrality in the graph, community detection

## Abstract

The number of metal-organic frameworks (MOF) as well as the number of applications of this material are growing rapidly. With the number of characterized compounds exceeding 100,000, manual sorting becomes impossible. At the same time, the increasing computer power and established use of automated machine learning approaches makes data science tools available, that provide an overview of the MOF chemical space and support the selection of suitable MOFs for a desired application. Among the different data science tools, graph theory approaches, where data generated from numerous real-world applications is represented as a graph (network) of interconnected objects, has been widely used in a variety of scientific fields such as social sciences, health informatics, biological sciences, agricultural sciences and economics. We describe the application of a particular graph theory approach known as social network analysis to MOF materials and highlight the importance of community (group) detection and graph node centrality. In this first application of the social network analysis approach to MOF chemical space, we created MOFSocialNet. This social network is based on the geometrical descriptors of MOFs available in the CoRE-MOFs database. MOFSocialNet can discover communities with similar MOFs structures and identify the most representative MOFs within a given community. In addition, analysis of MOFSocialNet using social network analysis methods can predict MOF properties more accurately than conventional ML tools. The latter advantage is demonstrated for the prediction of gas storage properties, the most important property of these porous reticular networks.

## 1. Introduction

The modelling and examination of complex systems that contain chemical, biological, ecological, economic, social, technological, and other types of information is a very challenging process if the number of elements in the system becomes very large. Metal-Organic Frameworks (MOFs), a class of chemical compounds composed of metal nodes connected via organic linker molecules, represent a particularly complex example from materials science. The wide variety of metal nodes and organic linker molecules suitable for MOF synthesis and the virtually unlimited number of linker/node combinations lead to an enormous size of the MOF chemical space. While the number of experimentally characterized MOFs already exceeds 100,000 [1], there is no upper limit for the total number of these reticular networks. This diversity in MOF chemistry makes it extremely difficult to navigate through the large design space and to identify MOF materials with suitable properties for a desired application or to identify most representative MOFs for an anticipated study, that cover best the available design space. Identifying an appropriate MOF for a given application in this huge chemical space can, in principle, be carried out by high throughput screening of existing or hypothetical MOFs databases. This screening can be carried our either experimentally or theoretically [2]. However, this approach becomes extremely costly with increasing size of the database. Recently, new paradigms for the discovery and rational design of materials have been established that are based on Machine Learning (ML) as well as sophisticated data science analysis methods and algorithms [3]. In the field of MOFs, first examples, where ML methods were employed to predict material properties or even to design new MOF structures and predict synthesis conditions have already been successfully demonstrated [4].

The simulation of the adsorption capacity for gases, probably the most important property of MOFs for existing applications, provides an interesting example of ML-based strategies for handing materials classes with large size. The presently most popular ML algorithms are Support Vector Machine, Random Forest and Neural Networks for predicting the absorption of guest molecules by MOFs [5]. In addition, deep learning, a particularly effective ML algorithm, has been used in a number of different applications [6]. For many practical applications, the stability of MOFs in aqueous environments is an important prerequisite and ML-based models could accurately predict water stability of MOFs [7].

When applying ML models, a first important step is the selection of descriptors. The number of the different parameters describing MOF structures is simply too large for a direct, straightforward analysis, therefore also ML-based workflows were introduced, that allow extracting the most valuable descriptors within a given family of MOFs [8]. Combining data mining and machine learning has allowed for the prediction of MOF synthesis [9] and MOF stability [10]. In another study, the authors proposed a machine-learning algorithm to predict the possibility of metal-linker combinations for the guest accessibility of MOFs. In this method, various ML models were evaluated to learn the connection between component chemistry and MOF properties without explicitly requiring a priori knowledge of the MOF structure [11].

In this work, we propose a new approach based on the social network analysis for analysing the chemical space of metal organic frameworks. Social network analysis was originally established in the field of social sciences [12,13], but has been expanded to virtual learning [14], health informatics [15,16,17,18], life sciences [19,20,21,22], agriculture [23,24,25], economy [25,26], and others.

Building a social network enables the use of machine learning techniques based on graph mining to extract valuable knowledge from the MOFs data. Our goal in this study is to demonstrate that "social networks" constructed from MOFs are a valuable tool for analysing large MOFs databases, allowing to navigate through the MOF chemical space, identify suitable MOFs for a given application or desired study, and to curate large datasets efficiently. 

We used social network analysis (SNA), rather than more traditional machine learning algorithms, since SNA outperforms other ML models in visualizing complex relationships between different MOFs. Additionally, SNA allows to extract information about the properties of a given (e.g., so far unknown) MOF by its relationship to “neighbouring” (known) MOFs in the social graph. SNA therefore allows to extract useful information, e.g., find the most representative MOFs or identify implicit and hidden dependencies between MOFs.

Two primary types of SNA were performed as part of the current research: centrality and community detection. In the centrality analysis, parameters are determined that measure the characteristics of a given MOF node in the graph in relation with other MOF nodes (in this case other MOFs) in the graph. In MOFSocialNet, we deal with different types of centralities, degree centrality, and closeness centrality. Degree centrality focuses on the links of one MOF node to other MOF nodes. MOF nodes with a high degree centrality can be regarded as important MOF structures with similar characteristics to many other MOF structures in the dataset. The closeness centrality is computed by considering the average distance from the target MOF node to the other MOF nodes in the networks. MOF nodes with a high degree centrality can be regarded as very representative MOF structures for the given set of analyzed MOF structures [27]. Node centralities allow to identify the most important or influential node in a graph. For instance, a high value of degree centrality identifies nodes that are in the middle of the network. Thus, by blending the information provided by the different centralities allows to analyze the MOF networks and to find correlations between MOFs.

One other parameter, which we looked at in MOFSocialNet, is community detection [28,29,30,31,32,33,34]. Community detection is essentially a type of clustering problem. Community detection aims to group the nodes according to the relations between them to form strongly related sub-graphs from the entire graph. For example, detection of communities holds an important place in the analysis and functional prediction of the interaction networks between proteins and other molecules in biological cells [21] or to predict and identify disease genes.

In the context of MOFSocialNet, community detection can be applied to provide an overview and a structure to highly diverse MOF datasets. Social network analysis can therefore help to rationalize the categories used to describe MOF types, moving away from “most popular MOF types” towards categories based on more objective features or properties of the MOFs.

## 2. Methods

**Figure 1:**
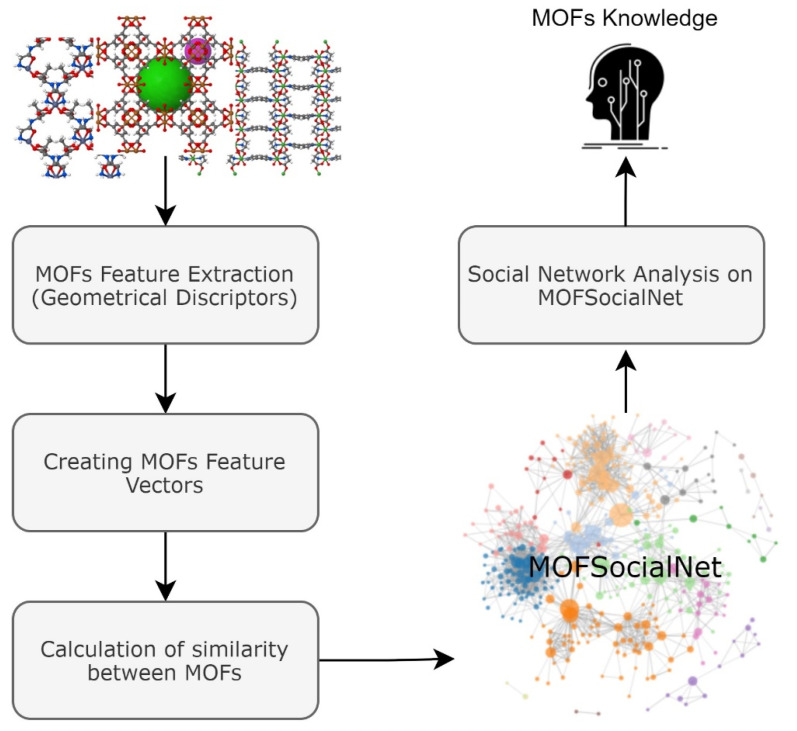
Workflow for the MOFSocialNet creation and analysis.

In this paper, we construct a social network called MOFSocialNet from geometrical descriptors of MOFs in the CoRE-MOFs database. MOFSocialNet is an undirected, weighted, and heterogeneous social network. Following the construction of MOFSocialNet, we provide a set of social network analytic processes to extract valuable knowledge from the MOF data using graph-mining algorithms. The full workflow of the MOFSocialNet is shown in Figure 1. In the first step, we created a feature vector for each MOF. In the following step, the similarity between each pair of MOF vectors is calculated based on vector similarity methods. In this step we created the MOFs social graph, named MOFSocialNet, where MOFs are the nodes and the similarity between the MOF feature vectors are the links (i.e., the relationships between MOFs). Finally, after removing irrelevant links in the graph, we applied social network analysis methods to extract valuable knowledge from this graph. In subsequent sections, all steps are described in detail.

### 2.1. Creating MOF Feature Vectors

A feature vector is an n-dimensional vector of numerical features that can e.g., describe an object in pattern recognition using machine learning. In the case of MOFs, every property depends on a set of specific descriptors, i.e., geometrical, chemical, topological, and energy-based descriptors [35]. Before applying the data analysis process, it is critical to identify key descriptors that are highly correlated with the property of interest of the MOF. 

For a demonstration of the proposed approach, we limited ourselves to a subset of 1000 MOFs in the CoRE-MOFs database and used eight geometric descriptors for MOF (see Table 1). Therefore, each MOF is assigned to an eight-dimensional feature vector. 

In order to simplify the further analysis, we normalized the values of the numerical columns in the dataset to a common scale. The most common method of data normalization is Min-Max normalization, which values are transformed into decimals between 0 and 1, using formula 1:

(1) $$v^{\prime }=\frac{v-min_{A}}{max_{A}-min_{A}}$$

where $min_{A}$ and $max_{A}$ denote the minimum and maximum values of the corresponding property *A*. In the following, the original and normalized values are denoted by v and $v^{\prime }$, respectively. As a consequence, all *v*′ adopt values from the intervall (0,1) [36].

### 2.2. Constructing MOFSocialNet

We define MOFSocialNet as a graph G = (V,E) [17], where V = {v1, v2, …, vn} is the vertex set, and E = {eij = edge from vi to vj|1 ≤ i, j ≤ n, i ≠ j} is an edge set.

For the construction of the graph, a metric must be introduced to measure the distance between two vertices. In previous works, either direct (Euclidean or Manhattan metrics) or more invoked methods like Pearson’s product-moment correlation coefficient (PPMCC) or Cosine methods have been used.

In the current study, we have used the so-called cosine metrics. Given two vectors of MOF descriptors, $\bar{A}$ and $\bar{B}$, the cosine similarity, cos(θ), is computed as

(2) $$\mathrm{Similarity}=\cos(\theta )=\frac{\sum_{i=1}^{n}A_{i}B_{i}}{\sqrt{\sum_{i=1}^{n}A_{i}^{2}}\sqrt{\sum_{i=1}^{n}B_{i}^{2}}}$$

where A_i_ and B_i_ are the components of the feature vectors $\bar{A}$ and $\bar{B}$, respectively. The resulting similarity ranges adopt values from the interval (0,1), with 0 signifying the dissimilarity of the MOFs. A value of 1 reveals that the MOFs A and B are identical.

With this metric, now a graph representing all ~1000 elements of the database can be constructed, consisting of 1000 vertices and 22,000 edges.

In this initial graph, the number of edges is too large for an efficient analysis and an effective strategy to remove weak links must be introduced. Therefore, we removed all edges with a length less than a threshold parameter d.

**Figure 2:**
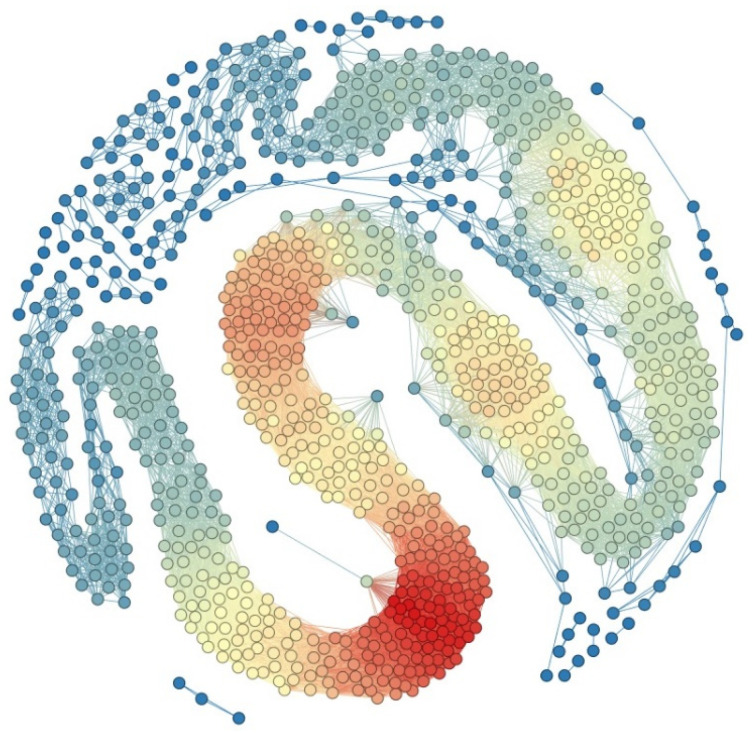
MOFSocialNet with 1000 Metal-Organic Frameworks, the visualization of the MOFSocialNet with Fruchterman Reingold layout in the Gephi software. Different colors indicate MOFs with a similar number of connections to other MOFs.

The representation of the sample network of MOFSocialNet after elimination of weak links using a value for d = 0.9999 (reducing the number of nodes to 2214) is shown in Figure 2. The presence of an edge between two MOFs thus indicates that the similarity is above the threshold value.

**Figure 3:**
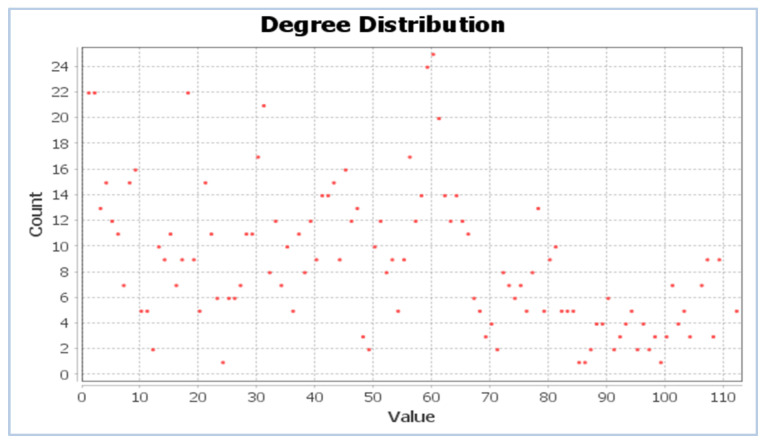
Degree distribution of the MOFSocialNet—the degree distribution is the probability distribution of node degrees over the whole network.

An important property of a particular node is its degree, the number of edges linking it to other nodes. The probability distribution of these degrees over the whole network is shown in Figure 3. The average degree based on the diagram is 46.

### 2.3. Centrality Measures in MOFSocialNet

For the next step of the analysis, we determined the centrality of nodes. Centrality means the relative significance of nodes (or vertices) and links (or edges). In our context, centrality measures how similar a MOF is to other MOFs within MOFSocialNet.

The simplest approach is degree centrality, which for a given node is obtained by counting the number of links connecting to other nodes. In MOFSocialNet, a MOF with a high degree centrality has very similar properties to many other MOFs. For the MOFSocialNet graph (G), degree centrality (*C_d_*) is defined as:

(3) $$C_{d}=\frac{k_{i}}{n-1}$$

where *k_i_* is the degree (number of edges connected to a node) of node *i* and *n* is the total number of nodes in the graph [35].

**Figure 4:**
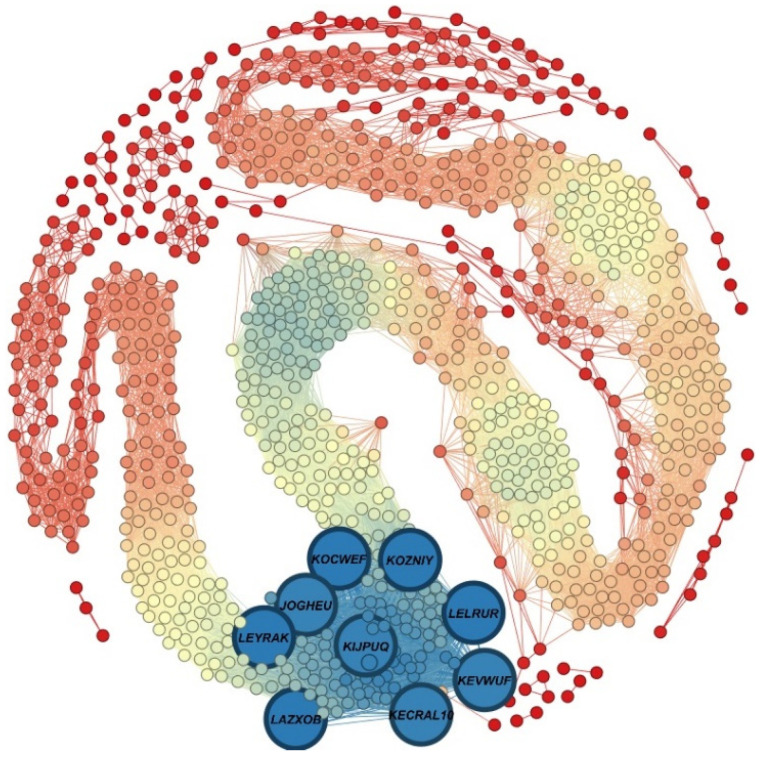
Visualization of MOFSocialNet with the top 10 MOFs with the highest degree, drawn with Fruchterman Reingold layout in the Gephi software. The blue nodes in bold indicate the MOFs with the highest degree.

Table 2 presents the 10 MOFs with highest degree and Figure 4 illustrates the MOFSocialNet with these MOFs being highlighted.

The closeness centrality (*C_c_*) of a vertex is a measure of the closeness of the vertex to the rest of the vertices in a graph. The *C_c_* of a vertex is computed as the inverse of the sum of the hop counts (farness) of the shortest paths from the vertex to the rest of the vertices in the graph. If *d*(*i*, *j*) is the geodesic distance between two vertices v_i_ and v_j_ in a graph, then the closeness centrality of a vertex v_i_ could be computed as the sum of the geodesic distances to the vertices v_j_ that are in the same component as v_i_ (a component is the largest set of vertices that are reachable from each other) [37].

Closeness centrality (*C_c_*) is defined as:

(4) $$C_{c}=\frac{n-1}{\sum_{j}d\left(i,j\right)}$$

where *i* is the starting node, *j* the target node, and *d*(*i*, *j*) is the distance between them. This measures the distance from the starting node to other nodes in the graph [38].

**Figure 5:**
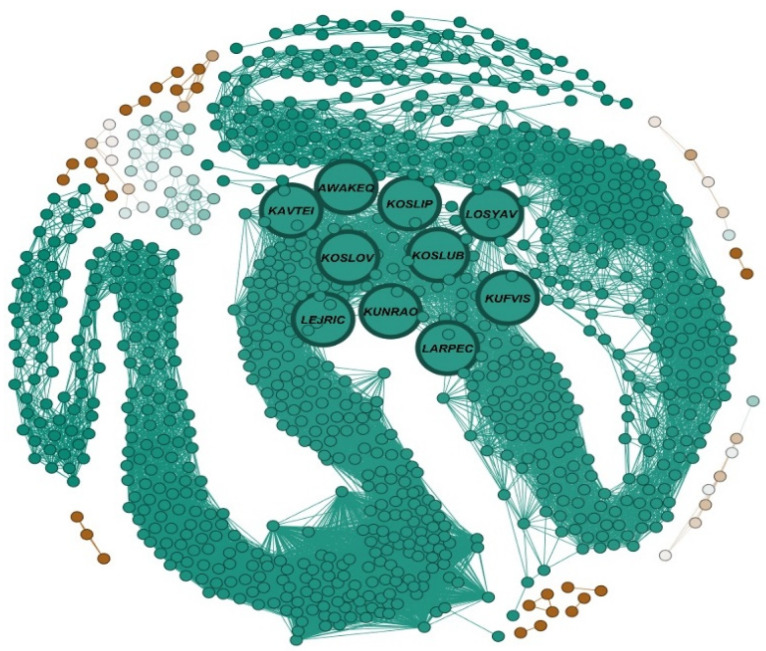
Visualization of the MOFSocialNet with the top 10 MOFs with the highest closeness centrality, drawn with Fruchterman Reingold layout in the Gephi software. The nodes in bold indicate the MOFs with the highest closeness centrality.

The closeness centrality captures the accessibility of network components. In the MOFSocialNet, being a network of MOF structures, closeness centrality can identify the most representative MOFs of a given dataset. Table 3 and Figure 5 illustrate the network, highlighting the ten MOFs with the highest closeness centrality.

### 2.4. Community Detection in MOFSocialNet

At the most abstract level, given a Social network G = (V,E), a community can be defined as a subgraph of the network including a set VC ⊆ V of Social Network entities that are associated with a common element of interest. This element can be a topic, a person of the real world, a place, an event, an activity, or a material such as metal organic framework.

Community detection is a common method in the social graph to categorize a large graph in sub-group with similar features or properties. Methods and algorithms exist for community detection in social networks. In this paper, we used a Louvain community detection algorithm to effectively extract communities. In the Louvain Method of community detection, first small communities are found by optimizing modularity locally on all nodes, then each small community is grouped into one node and the first step is repeated [39].

**Figure 6:**
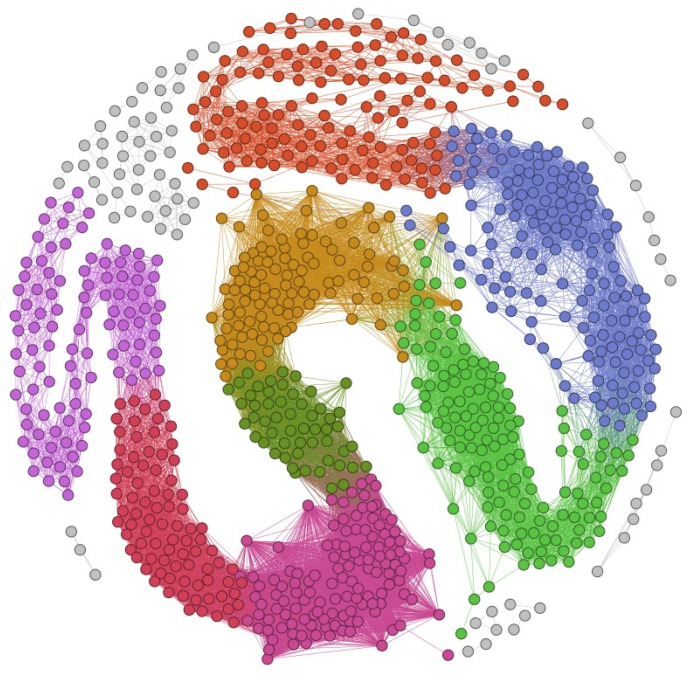
Visualization of MOFs communities in the MOFSocialNet. The node colors represent the community nodes, which are derived from the Lovain method. Nodes of similar color form a community. The graph is shown with the Fruchterman Reingold layout in the Gephi software.

In MOFSocialNet, 24 communities were identified through the Lovain method. In Figure 6, we visualized the graphs of the 24 communities. To improve the readability of the graph, we removed the MOFs node labels. It should be noted that using the Gephi software, a further round of community detection was carried out on each of the 24 communities using the Louvain algorithm. The colors of the graph indicate the different communities. Thus, the nodes of the same color belong to the same community.

One metric to evaluate the extracted community is modularity. Modularity measures how strongly a network can be divided into different communities. Networks with high modularity have dense connections between the nodes within each community but sparse connections between nodes in different communities [39]. The modularity value is in the range of −0.5 to 1. A value of 1 indicates the highest modularity, the modularity of the MOFSocialNet with 1000 MOFs is 0.748. Table 4 lists the different MOFs communities depicted in Figure 6, indicates the number of MOF nodes in each community and the MOF with highest centrality in each community.

### 2.5. Application of MOFSocialNet to Predict the Crystal Density of Unknown MOFs

To evaluate whether properties of new MOFs can be predicted using MOFSocialNet, we randomly chose three MOFs from the CoRE MOFs database. For these MOFs, we excluded the crystal density as input during featurization and placed the MOFs within the MOFSocialNet. We then predicted the crystal density of the new MOFs by simply averaging the crystal density of the ten nearest neighbors. The results show an outstanding prediction accuracy for the crystal density of 99.69% for MOF ABAYOU, 99.79% for ABIXOZ, and 99.96% for ACOLIP. Table 5 presents the density range of the MOFs in the communities.

### 2.6. Use of MOFSocialNet to Predict Gas Adsorption in the Metal Organic Framework

In the final part of our investigation, we evaluated how well the communities extracted from MOFSocialNet can be exploited for predicting gas adsorption properties of MOFs for CO_2_ and CH_4_. To evaluate the performance, we compared the prediction performance of MOFSocialNet with three common ML models, namely K Nearest Neighbour (KNN), Gradient Boosting Regression, and Deep Learning. All ML predictions were performed using Rapidminer Machine Learning tools. The efficiency of each ML algorithm was assessed by computing with the mean absolute error (MAE) which is given in Equation (5).

(5) $$MAE=\frac{\sum_{i=1}^{n}\left|y_{i}-x_{i}\right|}{n}$$

where $y_{i}$ is the prediction value of gas adsorption and $x_{i}$ the true value of respected gas adsorption.

**Figure 7:**
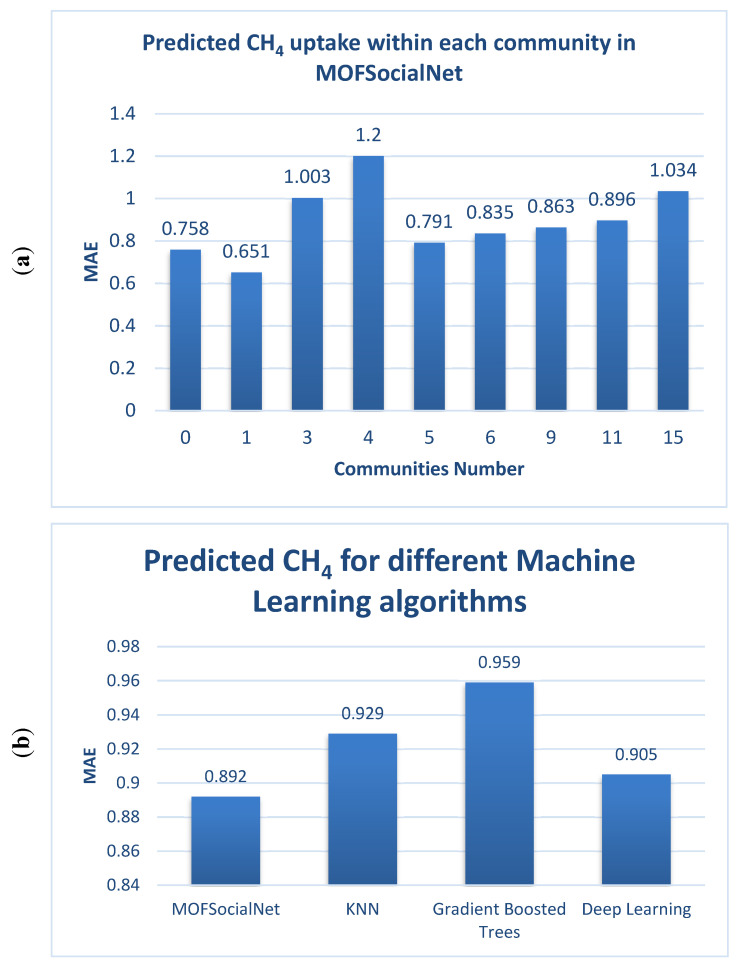

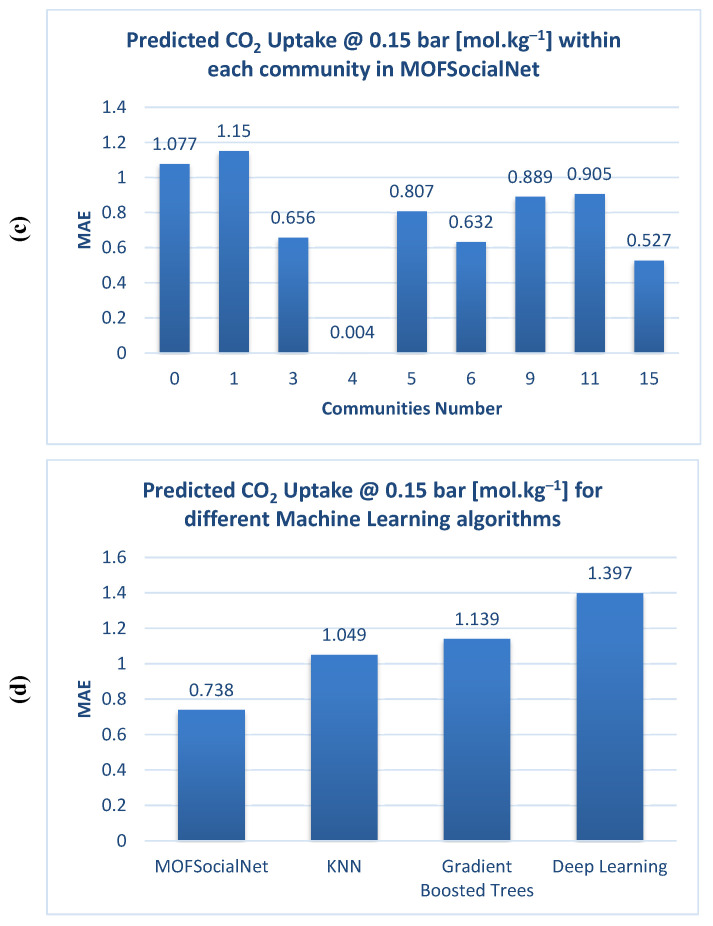
(**a**) Prediction of CH_4_ adsorption within each community in MOFSocialNet; (**b**) Average prediction of CH_4_ uptake for all communities in MOFSocialNet compared with the prediction of other machine learning algorithms; (**c**) Prediction of CO_2_ uptake @ 0.15 bar (mol∙kg^−1^) within each community in MOFSocialNet; and (**d**) Average prediction of CO_2_ uptake @ 0.15 bar (mol∙kg^−1^) for all communities in MOFSocialNet compared with the prediction of other machine learning algorithms. Only communities with a reasonable number of MOFs to predict are depicted in this figure.

The gas adsorption method by MOFSocialNet was performed by (a) creating the MOFSocialNet, as explained in the previous section, (b) extracting communities from MOFSocialNet, and (c) predicting gas adsorption in each individual community. The MAE performance parameter for each individual community is presented using the prediction of CO_2_ absorption. Moreover, the overall MAE results were compared to the three main well-known algorithms. Similar to CO_2_ prediction, the prediction of gas adsorption for CH_4_ is presented for each community, and the overall MAE results were compared to the three main well-known algorithms. Using MOFSocialNet significantly improved the prediction compared to the reference ML algorithms, as shown in the results in Figure 7.

The findings indicate a significant improvement in the prediction presented in Table 6. 

## 3. Conclusion and Future Direction

In this paper, we demonstrated that social network analysis (SNA), a tool developed in the social sciences, is well suited to analyse MOF structural databases. The MOFSocialNet was constructed using geometrical descriptors provided in an existing MOF database, CoRE-MOF, yielding an undirected, weighted, and heterogeneous social network. We then used MOFSocialNet as a new tool to guide MOF researchers through the vast chemical space of existing and hypothetical MOFs. For demonstration, we employed SNA to identify the most representative MOFs in this set of research data and to detect MOFs communities, i.e. families of MOFs with similar properties. Furthermore, within each community, the SNA identifies the most representative MOFs structure. Our approach can help to rationally select the most appropriate MOF structures for future studies, such as the most promising or diversified MOF. To demonstrate the feasibility of property prediction via SNA we trained communities extracted from MOFSocialNet, then predicted CO_2_ and CH_4_ adsorption and evaluated the accuracy of the prediction. Interestingly, SNA outperformed three common ML models, namely K Nearest Neighbour (KNN), Gradient Boosting Regression, and Deep Learning. The proposed SNA approach can accelerate the analysis of MOFs structure, even by increasing the amount of theoretical and experimental data on MOFs. MOFSocialNet as a novel framework can be extended to processing and curating large MOFs databases.

## Figures and Tables

**Table 1:** Geometry Descriptors in CoRE-MOFs Database.

Name of the Descriptors	Symbol
Sphere	Di
Largest free sphere	Df
Largest included sphere along free path	Dif
Crystal density	*ρ*
Volumetric surface area	VSA
Gravimetric surface area	GSA
Volumetric pore volume	VPOV
Gravimetric pore volume	GPOV

**Table 2:** Ten MOFs with highest degree.

No.	Name of the MOF	Degree
1	LELRUR	112
2	LAZXOB	112
3	KOCWEF	112
4	KOZNIY	112
5	LEYRAK	112
6	j.ica.2016.05.002_2	109
7	JOGHEU	109
8	KEVWUF	109
9	KIJPUQ	109
10	KECRAL10	109

**Table 3:** Ten MOFs with highest closeness centrality.

No.	Name of the MOF	Closeness
1	KOSLIP	0.122975
2	KUFVIS	0.122958
3	LARPEC	0.122584
4	KOSLUB	0.122398
5	KOSLOV	0.122331
6	LOSYAV	0.122213
7	KAVTEI	0.122179
8	LEJRIC	0.122179
9	KUNRAO	0.122163
10	AWAKEQ	0.122028

**Table 4:** Community descriptions in the MOFSocialNet.

Community	MOF with Highest Centrality in Community	No. of MOF	Community	MOF with Highest Centrality in Community	No. of MOF	Community No.	MOF with Highest Centrality in Community	No. of MOF	Community	MOF with Highest Centrality in Community	No. of MOF
**1**	j.jlumin.2016.09.057_ mmc2	**54**	**7**	KITCAT	**139**	**13**	j.matlet.2016.01.080_ mmc1	2	19	KICXAX	**2**
**2**	KUYPIF	**154**	**8**	KUNRAO	**103**	**14**	JODGEP	3	20	KIYMIP	**2**
**3**	KALGEL	**84**	**9**	ACALIB	**2**	**15**	KUDWAJ	100	21	KOJZEP	**2**
**4**	ABESUX	**3**	**10**	KUVMIZ	**8**	**16**	KABXAO	7	22	KONFIE	**2**
**5**	AXIPEE	**128**	**11**	AVETAY	**125**	**17**	KARNAU	3	23	KUGCOF	**2**
**6**	LELDOX	**22**	**12**	acsami.6b11116_ am6b11116_si_002	**5**	**18**	JUFREJ	10	24	JOGSAA	**5**

**Table 5:** Density range of the MOFs in the communities.

Community Number	Density (g/cm^3^) Range	
8	0.380351	0.334035
14	0.43704	0.402001
18	0.462868	0.458126
21	0.514247	0.506406
10	0.554774	0.536397
4	0.674371	0.58881
15	0.918625	0.697914
1	1.01242	0.926612
9	1.08519	1.01371
0	1.13994	1.08774
5	1.23288	1.141
20	1.35327	1.34717
11	1.42464	1.22885
12	1.42645	1.41674
6	1.68807	1.43058
7	1.78668	1.76873
13	2.17773	2.16342
3	2.25323	1.58742
22	2.25449	2.20818
16	2.5731	2.30777
23	2.84923	2.83999
17	3.02612	2.73182
19	3.58491	3.50049
2	3.8283	3.69928

**Table 6:** Evaluation of predictive performance of CO_2_ and CH_4_ using MOFSocialNet and compared reference machine learning algorithms.

Method Name	MAE for CO_2_ Prediction	MAE for CH_4_ Prediction
KNN	1.049	0.929
Gradient Boosted Trees	1.139	0.959
Deep Learning	1.397	0.905
**MOFSocialNet**	**0.738**	**0.892**

## Data Availability

The data presented in this study are openly available in [materialscloud] at [https://doi.org/10.24435/materialscloud:3y-gr], accessed on 28 January 2022, reference number [40].
